# Association between Low Fruit and Vegetable Consumption and Colorectal Polyps in Thailand

**DOI:** 10.31557/APJCP.2020.21.9.2733

**Published:** 2020-09

**Authors:** Kannika Supachai, Bunchorn Siripongpreeda, Kamonwan Soonklang, Napatsawan O-Pad, Kanlayanee Krohkaew, Chanisara Suebwongdit, Suchada Panaiem

**Affiliations:** 1 *Department of Nursing, Chulabhorn Hospital, HRH Princess Chulabhorn College of Medical Science, Chulabhorn Royal Academy, Bangkok, Thailand. *; 2 *Department of Surgery, Faculty of Medicine and Public Health, HRH Princess Chulabhorn College of Medical Science, Chulabhorn Royal Academy, Bangkok, Thailand. *; 3 *Data Management Unit, HRH Princess Chulabhorn College of Medical Science, Chulabhorn Royal Academy, Bangkok, Thailand. *

**Keywords:** Colorectal polyps, colorectal cancer, fruit and vegetable consumption

## Abstract

**Objective:**

This study aimed to evaluate the association between low fruit and vegetable consumption and colorectal polyps.

**Methods:**

A retrospective study was conducted among 1,228 participants aged 50-65 years who completed 2-time colonoscopy exams at the first and the fifth year of a colorectal screening program. Consuming less than one serving of fruit and vegetable daily was rated as low. Colorectal polyps from colonoscopy findings were recognized in 3 types: hyperplastic, low risk and high risk adenomatous polyps.

**Results:**

The findings demonstrated high prevalence of low fruit (93.6%) and low vegetable (85.8%) consumption. Exercising individuals were more likely to consume both fruit (OR 2.28, 95%CI 1.42-3.65) and vegetable (OR 1.40, 95%CI 1.00-1.96), while smoking history individuals tended to consume vegetable (OR 2.08, 95%CI 1.22-3.55). Low fruit consumption was strongly associated with high risk adenomatous polyps (OR 4.39, 95%CI 2.40-8.03), while low vegetable consumption was distinctively associated with low risk (OR 6.26, 95%CI 4.11-9.55) and high risk adenomatous polyps (OR 8.64, 95%CI 5.30-14.09).

**Conclusion:**

This study provides additional evidence of the association between low fruit and vegetable consumption and colorectal polyps. Enhancing people fiber eating behavior may help preventing colorectal cancer risk.

## Introduction

Minerals and fiber in fruit and vegetables have been scientifically proved as significant components of a healthy diet (Wallace et al., 2019). Appropriate amounts of fruit and vegetable consumption help prevent micronutrient deficiency and noncommunicable diseases (NCDs), such as cardiovascular diseases and certain cancers (World Health Organization: WHO, 2003). However, consumption of inadequate fruit and vegetable contributed to 3.9 million deaths globally in 2017 (WHO, 2019). Low fruit and vegetable consumption has been linked to NCDs and leads to premature death (Wang et al., 2014; Aune et al., 2017), as well as colorectal cancer risk (Vogtmann et al., 2013; Aoyama et al., 2014: Ben et al., 2015).

Although the cause of cancer development in deoxyribonucleic acid (DNA) remains unclear (Kang, 2011), colorectal cancer (CRC) is the sequence of colorectal polyps which grow on the inner surface of the colon. Colorectal polyps can be histologically classified as non-neoplastic and neoplastic tumors (Shussman and Wexner, 2014). Non-neoplastic polyps include hyperplastic, inflammatory and hamartomatous types. Neoplastic polyps include adenomas and serrated types. While non-neoplastic polyps typically do not turn to cancer, neoplastic polyps increase the risk of cancer. However, a study has proved that people with hyperplastic polyposis (multiple lesions) had a high risk of colorectal cancer (Hyman et al., 2004).

Factors associated with colorectal polyps include age, male sex, BMI (body mass index), heavy alcohol consumption, smoking, physical inactivity, lower educational attainment, lower socio-economic status and low fiber dietary consumption (Vogtmann et al., 2013; Ben et al., 2015; Schwingshackl et al., 2018). 

Low fruit and vegetable consumption is associated with CRC risk. While, fruit seems to have an inverse association with CRC risk, high vegetable consumption plays a stronger role in preventing the progression of colorectal adenoma. High total fruit and certain fruits and vegetables intake may reduce the risk of colorectal adenomas (Vogtmann et al., 2013; Ben et al., 2015). Moreover, low fruit and vegetable consumption is associated with NCDs: type 2 diabetes (Wu et al., 2015), hypertriglyceridemia and hyperlipidemia (Kjollesdal et al., 2016), hypertension (Li et al., 2016; Borgi et al., 2016) and cardiovascular disease risk (Aune et al., 2017).

Fruit and vegetable consumption is a dietary behavior of adulthood developing from habits cultivated during childhood (Othman et al., 2012). However, consuming low fruit and vegetable has been reported globally (Lee et al., 2015; Wallace et al., 2019). Only one third of the world population consumed fruit and vegetables following WHO recommendation (Msambichaka et al., 2018).

WHO recommends consuming at least 5 servings or 400 gram of fruit and vegetables daily (excluding potatoes and other starchy tubers) to prevent chronic diseases and provide several micronutrients the body needs (WHO, 2003). The 2015 to 2020 dietary guidelines for Americans recommend adults to consume 1.5 to 2 cups of fruits and 2 to 3 cups of vegetables daily (US Department of Health and Human Services and US Department of Agriculture, 2015).

The Thai national survey III found that people consumed fruits and vegetables less than recommended by average frequencies of fruit and vegetable consumption equal to 4.56 and 5.97 days per week. The daily numbers of servings of fruits, vegetables, and fruit plus vegetables were 1.46, 1.78 and 3.24 servings, respectively. Those varied by age, sex, marital status, living region, educational attainment and household income (Satheannoppakao et al., 2009).

Chulabhorn Hospital has launched a royal charity project involving a CRC screening program to prevent disease and promote health in Thai populations across the country. Five years follow-up of this project showed a high prevalence of colorectal polyps (Siripongpreeda et al., 2016). While adenomatous polyps are considered as precursors of colorectal cancer, the growth pattern and high grade of dysplasia are determined as the malignant potential (Shussman, 2014). However, a study reports that about 50% of polyps transform to malignancy (Conteduca et al., 2013). In addition, low fruit and vegetable consumption increases CRC risk (Vogtmann et al., 2013; Wang et al., 2014; Ben et al., 2015; Schwingshackl et al., 2018). Hence, this study aimed to evaluate the association between low fruit and vegetable consumption and colorectal polyps among participants of the colorectal cancer screening program. 

## Materials and Methods

A retrospective study was conducted to review data from the electronic case record forms of participants who joined the phase I of CRC screening program at Chulabhorn Hospital between 2009 and 2013. Inclusion criteria comprised adults aged 50 to 65 years who completed 2-time colonoscopy exams at the first and the fifth year following the protocol. Exclusion criteria included subjects having history of CRC and other types of cancers. This study was approved by the Human Research Ethics Committee of the Chulabhorn Research Institute (No.008/2020).

Data were collected based on literature review and existing data including personal characteristics (sex, age, marital status, educational attainment, income, and region of residence) and behavior (exercising, smoking history, and fruit and vegetable consumption). Moreover, type 2 diabetes, hyperlipidemia, and hypertension were identified as NCDs, and clinical findings from colonoscopy were assessed following types of colorectal polyps reported: hyperplasia, low risk adenomatous polyps and high risk adenomatous polyps. Based on NCCN guidelines, low risk refers to pathological size less than 1 cm and number of adenomas less than 3 with tubular or serrated or low grade dysplasia, while high risk refers to higher size and more numbers or showing growth patterns of tubulovillous or villous adenoma or high grade dysplasia (National Comprehensive Cancer Network, version 2. 2017). 

 According to the Thai national survey, the Thai population consumed fruit an average of 4.56 and vegetables 5.7 days per week and daily numbers of servings of fruit and vegetables averaged 1.46 and 1.78 (Satheannoppakao et al., 2009). Therefore, fruit and vegetable consumption less than seven servings weekly were rated as low. 

PASW statistics 18 was employed to analyze the data. Descriptive analysis was applied to describe the characteristics of participants. Binary logistic regression was performed to determine the factors governing fruit and vegetable consumption. Multinomial logistic regression was constituted to evaluate the association between low fruit and vegetable consumption and colorectal polyps.

## Results

In total 1,404 participants joined the CRC screening program, were instructed for bowel preparation. If unclear bowel was showed, colonoscopy was retaken. All 1,228 completed the colonoscopy protocol. The majority comprised males (69.5%) with a mean age of 56.9 years (SD+4.2), married (67.7%), graduated bachelor degree or higher (37.7%), and low income level (75.5%). About 40% of participants lived in Bangkok. Those subjects showed high levels of unhealthy behavior including low fruit (93.6%) and vegetable consumption (85.8%), and low exercising behavior (64.7%). However, no smoking history was high (88.4%). Participants living with NCDs comprised hyperlipidemia (41.2%), hypertension (35.9%), and type 2 diabetes (13.8%). 

This study showed the factors determining fruit and vegetable consumption included exercising and smoking history behavior. Individuals with usual exercising were more likely to consume both fruit (OR 2.28, 95%CI 1.42-3.65) and vegetable (OR 1.40, 95%CI 1.00-1.96), while individuals with smoking history tended to consume vegetable (OR 2.08, 95%CI 1.22-3.55). However, this study found that NCDs were unassociated with fruit and vegetable consumption (Table 1).

Regarding the association between low fruit and vegetable consumption and colorectal polyps, colorectal polyp free group was categorized as reference for analysis. When adjusted exercising, low fruit consumption was strongly associated with high risk adenomatous polyps (OR 4.39, 95%CI 2.40-8.03). While adjusted exercising and smoking history, low vegetable consumption was distinctively associated with low risk adenomatous polyps (OR 6.26, 95%CI 4.11-9.55) and high risk adenomatous polyps (OR 8.64, 95%CI 5.30-14.09) (Table 2).

**Table 1:** Participants’ Characteristics and Factors Influencing Fruit and Vegetable Consumption (n=1,228)

Characteristic	n (%)	FC	VC
		OR (95%CI)	
Sex			
Male	853 (69.5)	Ref.	Ref.
Female	375 (30.5)	0.72 (0.38-1.35)	0.81 (0.52-1.27)
Age (mean, +SD)	56.9 (+4.2)	0.99 (0.93-1.05)	0.99 (0.95-1.03)
Marital status			
Single	178 (14.5)	Ref.	Ref.
Married	831 (67.7)	2.71 (0.94-7.75)	0.75 (0.42-1.33)
Widowed/divorced	219 (17.8)	0.85 (0.45-1.60)	0.87 (0.54-1.38)
Educational attainment			
Primary level	304 (24.8)	Ref.	Ref.
Secondary-diploma level	461 (37.5)	1.53 (0.76-3.08)	1.16 (0.72-1.85)
Bachelor’s degree and higher	463 (37.7)	1.18 (0.68-2.05)	1.03 (0.69-1.54)
Income (THB/month)			
<25,000	927 (75.5)	Ref.	Ref.
>25,000	301 (24.5)	0.99 (0.56-1.76)	0.83 (0.54-1.27)
Region of residence			
Bangkok	490 (39.9)	Ref.	Ref.
Central	40 (3.3)	0.78 (0.33-1.87)	1.19 (0.63-2.25)
Northeast	73 (5.9)	1.36 (0.57-3.23)	1.44 (0.75-2.77)
North	123 (10.0)	1.40 (0.52-3.82)	1.96 (0.94-4.06)
East	67 (5.5)	0.83 (0.17-4.20)	1.42 (0.50-4.04)
West	118 (9.6)	1.17 (0.36-3.82)	0.99 (0.39-2.53)
South	317 (25.8)	1.01 (0.29-3.57)	1.09 (0.43-2.79)
Exercising			
No	795 (64.7)	Ref.	Ref.
Yes	433 (35.3)	2.28 (1.42-3.65)	1.40 (1.00-1.96)
Smoking history			
No	1085 (88.4)	Ref.	Ref.
Yes	143 (11.6)	1.48 (0.67-3.26)	2.08 (1.22-3.55)
NCDs			
Type 2 diabetes	170 (13.8)	0.80 (0.36-1.79)	0.97 (057-1.63)
Hyperlipidemia	506 (41.2)	0.91 (0.54-1.53)	0.71 (0.49-1.02)
Hypertension	441 (35.9)	0.72 (0.41-1.26)	0.99 (0.69-1.44)
Fruit consumption			
<7 servings/wk	1149 (93.6)	-	-
>7 servings/wk	79 (6.4)	-	-
Vegetable consumption			
<7 servings/wk	1054 (85.8)	-	-
>7 servings/wk	174 (14.2)	-	-
Colorectal polyp types			
Hyperplastic	118 (9.6)	-	-
Low-risk adenoma	151 (12.3)	-	-
High-risk adenoma	95 (7.7)	-	-

FC, fruit consumption; VC, vegetable consumption

**Table 2:** Association between Low FV Consumption and Colorectal Polyps

Polyp types	Consumption	B	SE	Wald	df	Sig	Exp (B)	95%CI
Hyperplasia	FC<7 s/w	0.46	0.38	1.47	1	0.23	1.59	0.75-3.37
	VC<7 s/w	0.46	0.31	2.13	1	0.14	1.58	0.85-2.92
Low risk	FC<7 s/w	0.21	0.38	0.31	1	0.58	1.23	0.59-2.60
	VC<7 s/w	1.83	0.21	72.83	1	<.001	6.26	4.11-9.55
High risk	FC<7 s/w	1.48	0.31	22.98	1	<.001	4.39	2.40-8.03
	VC<7 s/w	2.15	0.25	74.95	1	<.001	8.64	5.30-14.09

The reference category is colorectal polyp free group; FV, fruit and vegetable; FC, fruit consumption; VC, vegetable consumption; s/w, servings per week

## Discussion

This study highlights the association between low fruit and vegetable consumption and colorectal polyps among participants of the CRC screening program. Though Thailand is a source of many kinds of tropical fruits and vegetables, and is named as a kitchen of the world; however, the prevalence of low dietary fiber is high. Only 6.4% of participants consumed fruits and 14.2% consumed vegetables at least one serving daily. The prevalence of low fruit and vegetable consumption does not differ from the global situation (Wallace et al., 2019; Lee-Kwan et al., 2017; Msambichaka et al., 2018; Szucs et al., 2017).

While related studies have shown that people with high socio-economic status were more likely to consume fruit and vegetable daily (Wang et al., 2014; Schwingshackl et al., 2018; Msambichaka et al., 2018), this study showed that high education and affordability did not influence fruit and vegetable consumption. Only the healthy behavior of exercising determined fruit and vegetable consumption. However, smoking history was linked to vegetable consumption. 

For decades, new trends of healthier behaviors have been promoted to prevent and control chronic diseases (Klein et al., 2014). However, fruit and vegetable consumption is a result of childhood preferences. One study showed that not only individual factors (sex, age, knowledge, self-efficacy, taste preference, outcome expectations/expectancies, skills in preparing fruits and vegetables), but also social factors (modeling, family support, family meals, peer influence), and environmental factors (income, occupational status, parents’ education, household availability, school availability, neighborhood, television viewing) were linked to fruit and vegetable consumption (Al-Otaibi, 2015). Moreover, fruit and vegetable consumption is an essential component of traditional diet that affects people fiber dietary lifestyle (Morgan et al., 2016).

In the light of the association between low fruit and vegetable consumption and adenomatous polyps, this study showed strong association between low fruit and vegetable consumption with two types of adenomatous polyps: low and high risk. The finding supported the association between low fruits and vegetable consumption and adenomatous polyps (Tantamango et al., 2011), while one meta-analysis showed weak association between vegetable consumption and CRC risk (Kashino et al., 2015). The previous study showed citrus, watermelon, and legumes group were inversely associated with CRC risk (Vogtmann et al., 2013). However, this study lacked of data specific fruits and vegetables for analysis.

Strengths of the current study included using large numbers of participants and types of colorectal polyps from clinical colonoscopy findings. However, limitations of our study included the absence of specific types of fruits and vegetables, and frequency and quantity of daily servings.

In conclusion, our study provides additional evidence of the association between fruit and vegetable consumption and colorectal polyps. While low fruit consumption associated with high risk adenomatous polyp, low vegetable consumption associated with both low and high risk adenomatous polyps. Enhancing people fiber dietary consumption from childhood should be set as a national policy to prevent CRC risk. 

## References

[B1] Al-Otaibi HH, Ostojic SM (2015). Factors influencing fruit and vegetable intake in adolescents.

[B2] Aoyama N, Kawado M, Yamada H (2014). Low intake of vegetables and fruits and risk of colorectal cancer: the Japan collaboration cohort study. J Epidemiol.

[B3] Aune D, Giovannucci E, Boffetta P (2017). Fruit and vegetable intake and the risk of cardiovascular disease, total cancer and all-cause mortality-a systematic review and dose-response meta-analysis of prospective studies. Inter J Epidemiol.

[B4] Ben Q, Zhong J, Liu J (2015). Association between consumption of fruits and vegetables and risk of colorectal adenoma: A PRISMA-compliant meta-analysis of observational studies. Medicine.

[B5] Borgi L, Muraki I, Satija A (2016). Fruit and vegetable consumption and the incidence of hypertension in three prospective cohort studies. Hypertension.

[B6] Conteduca V, Sansono D, Russi S (2013). Precancerous colorectal lesions (review). Int J Oncol.

[B7] Hyman NH, Anderson P, Blasyk H (2004). Hyperplastic polyposis and the risk of colorectal cancer. Dis Colon Rectum.

[B8] Kang GH (2011). Four molecular subtypes of colorectal cancer and their precursor lesions. Arch Pathol Lab Med.

[B9] Kashino I, Mizoue T, Tanaka K (2015). Vegetable Consumption and Colorectal Cancer Risk: An Evaluation Based on a Systematic Review and Meta-Analysis Among the Japanese Population. Jpn J Clin Oncol.

[B10] Kjollesdal M, Htet AS, Stigum H (2016). Consumption of fruits and vegetables and associations with risk factors for non-communicable diseases in the Yangon region of Myanmar: a cross-sectional study. BMJ Open.

[B11] Klein WMP, Bloch M, Hesse BW (2014). Behavioral research in cancer prevention and control: a look to the future. Am J Prev Med.

[B12] Lee-Kwan SH, Moore LV, Blanck HM (2017). Disparities in State-Specific Adult Fruit and Vegetable Consumption - United States, 2015. MMWR.

[B13] Li B, Li F, Wang L (2016). Fruit and vegetables consumption and risk of hypertension: a meta-analysis. J Clin Hypertension (Greenwich).

[B14] Morgan EH, Vatucawaqa P, Snowdon W (2016). Factors influencing fruit and vegetable intake among urban Fijians: A qualitative study. Appetite.

[B15] Msambichaka B, Eze IC, Ramadhani Abdul ID (2018). Insufficient fruit and vegetable intake in a low- and middle-income setting: a population-based survey in semi-urban Tanzania. Nutrients.

[B16] National Comprehensive Cancer Network (2017). Colorectal cancer screening. (Version 2.2017).

[B17] Othman KI, Karim MSA, Karim R. et al (2012). Factors influencing fruits and vegetables consumption behavior among adults in Malaysia. J Agribusiness Marketing.

[B18] Satheannoppakao W, Aekplakorn W, Pradipasen M (2009). Fruit and vegetable consumption and its recommended intake associated with sociodemographic factors: Thailand National Health Examination Survey III. Pub Health Nutr.

[B19] Schwingshackl L, Schwedhelm C, Hoffmann G (2018). Food groups and risk of colorectal cancer. Int J Cancer.

[B20] Shussman N, Wexner SD (2014). Colorectal polyps and polyposis syndromes. Gastroenterol Rep.

[B21] Siripongpreeda P, Mahidol C, Dusitanon N (2016). High prevalence of advanced colorectal neoplasia in the Thai population: a prospective screening colonoscopy of 1 404 cases. BMC Gastroenterol.

[B22] Szucs V, Guine R, Leal M (2017). Dietary fibre: eating habits and knowledge in different regions of the globe. Millenium.

[B23] Tantamango YM, Knutsen SF, Lawrence Beeson WL (2011). Foods and food groups associated with the Incidence of colorectal polyps: the Adventist health study. Nutr Cancer.

[B25] Vogtmann E, Xiang YB, Li HL (2013). Fruit and vegetable intake and the risk of colorectal cancer: Results from the Shanghai Men’s Health Study. Cancer Causes Control.

[B26] Wallace TC, Bailey RL, Blumberg JB (2019). Fruits, vegetables, and health: a comprehensive narrative, umbrella review of the science and recommendations for enhanced public policy to improve intake. Crit Rev Food Sci Nutr.

[B27] Wang FW, Hsu PI, Chuang HY (2014). Prevalence and risk factors of asymptomatic colorectal polyps in Taiwan. Gastroenterol Res Pract.

[B28] Wang X, Ouyang Y, Liu J (2014). Fruit and vegetable consumption and mortality from all causes, cardiovascular disease, and cancer: systematic review and dose-response meta-analysis of prospective cohort studies. BMJ.

[B29] World Health Organization (2003). Diet, nutrition and the prevention of chronic diseases.

[B30] World Health Organization (2019). Increasing fruit and vegetable consumption to reduce the risk of non-communicable diseases; update 2019.

[B31] Wu Y, Zhang D, Jiang X (2015). Fruit and vegetable consumption and risk of type 2 diabetes mellitus: A dose-response meta-analysis of prospective cohort studies. Nutr Metabolism Cardiovascular Dis.

